# Evaluation of a Virtual Reality Percutaneous Nephrolithotomy (PCNL) Surgical Simulator

**DOI:** 10.3389/frobt.2019.00145

**Published:** 2020-01-14

**Authors:** Ben Sainsbury, Maciej Łącki, Mohammed Shahait, Mitchell Goldenberg, Amir Baghdadi, Lora Cavuoto, Jing Ren, Mark Green, Jason Lee, Timothy D. Averch, Carlos Rossa

**Affiliations:** ^1^Faculty of Science, Ontario Tech University, Oshawa, ON, Canada; ^2^Faculty of Engineering and Applied Science, Ontario Tech University, Oshawa, ON, Canada; ^3^University of Pittsburgh Medical Center, Pittsburgh, PA, United States; ^4^St. Michael's Hospital, Toronto, ON, Canada; ^5^University of Buffalo, Buffalo, NY, United States

**Keywords:** haptics, virtual reality, simulation, PCNL, percutaneous nephrolithotomy, surgical simulation and training

## Abstract

Percutaneous Nephrolithotomy is the standard surgical procedure used to remove large kidney stones. PCNL procedures have a steep learning curve; a physician needs to complete between 36 and 60 procedures, to achieve clinical proficiency. Marion Surgical K181 is a virtual reality surgical simulator, which emulates the PCNL procedures without compromising the well-being of patients. The simulator uses a VR headset to place a user in a realistic and immersive operating theater, and haptic force-feedback robots to render physical interactions between surgical tools and the virtual patient. The simulator has two modules for two different aspects of PCNL kidney stone removal procedure: kidney access module where the user must insert a needle into the kidney of the patient, and a kidney stone removal module where the user removes the individual stones from the organ. In this paper, we present user trials to validate the face and construct validity of the simulator. The results, based on the data gathered from 4 groups of users independently, indicate that Marion's surgical simulator is a useful tool for teaching and practicing PCNL procedures. The kidney stone removal module of the simulator has proven construct validity by identifying the skill level of different users based on their tool path. We plan to continue evaluating the simulator with a larger sample of users to reinforce our findings.

## 1. Introduction

**Figure 1:**
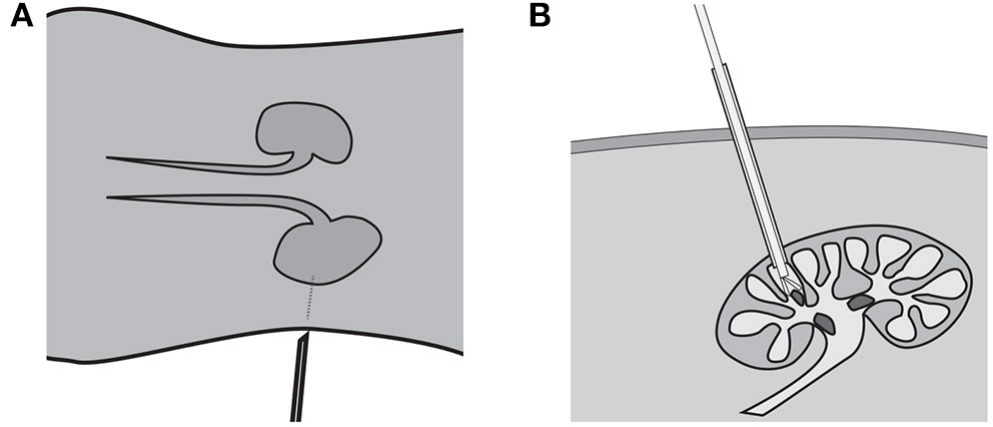
Kidney stone removal operation consists of two steps: establishing access to the kidney **(A)** shown in the coronal plane and removing the kidney stones shown in **(B)** from the sagittal plane.

Percutaneous Nephrolithotomy (PCNL) is the standard of care to treat kidney stones larger than 2 cm. The procedure involves two distinct steps. First, the surgeon establishes access to the kidney by puncturing the skin of the patient with a needle, like in Figure 1A. The location of the puncture is determined by using 2-dimensional fluoroscopic imaging to triangulate the location of the kidney. Once the access is established, the opening is dilated to allow the surgeon to insert a nephroscope with a grasper into the kidney, as shown in Figure 1B. Using the tools the stones are removed one at a time.

PCNL has a steep learning curve, requiring 36–60 cases to achieve competency and over 100 cases to achieve excellence. When Gill et al. (2012) looked into the operative experience of the urology trainee in the United Kingdom, they found that on average the trainee performed or assisted in only 19 PCNL's during their training period. In an attempt to standardize the minimum requirement to perform a safe PCNL (de la Rosette et al., 2008) showed that residents should perform 24 PCNL cases during training to a technical expertise level in PCNL; defined as an appropriate access and lithotripsy in simple complicated and medium complicated PCNL cases under strict supervision.

Urologists have introduced various simulation models to help trainees achieve competency level in a shorter length of time. Simulators used for assessment of PCNL skills include human cadavers, live animals, and Virtual Reality (VR) simulators to simulate human patients (Strohmaier and Giese, 2005; Knudsen et al., 2006; Mishra et al., 2010; Papatsoris et al., 2012; Zhang et al., 2013). Most of the literature on VR simulation is related to laparoscopic surgery and gastrointestinal endoscopy (Felsher et al., 2005). However, there is a paucity of data on the efficacy of training on VR simulators for endourologic skills, including PCNL. Three studies (Knudsen et al., 2006; Mishra et al., 2010; Papatsoris et al., 2012) showed the PERC Mentor simulator may improve the performance of the trainee. Virtual-reality-based simulators offer objective feedback, which often correlates positively with patient-related outcomes, and different case scenarios (Brydges et al., 2015). However, they often lack realistic haptic feedback which is key in improving laparoscopic skills of the trainee (Pinzon et al., 2016; Alleblas et al., 2017).

The Marion K181 PCNL Simulator is a virtual reality surgical simulator that can simulate the kidney access and stone removal procedures. It uses haptic force-feedback devices to render the interactions with the patient to the user and virtual reality headset to place the user in a virtual operating room.

This paper evaluates the face and construct validity of The Marion K181 PCNL Simulator. First, in section 2, we review various types of simulators available for resident training. We then describe the working elements of Marion Surgical K181 PCNL simulator in section 3. Section 4 presents the testing methodology and the results which are then discussed as pertaining to the face and construct validation of the simulator. Finally, in section 5 we describe the possible improvements to the simulator and our methodology. Let us begin by considering the current methods for training surgeons.

## 2. Current Training Methods

Surgical skills were traditionally self-taught through an apprenticeship model until 1904 when Halstead (1904) developed the rotating residency model. In this model, medical school graduates are placed in the hospital setting where they are given simple tasks initially. As they developed the necessary skills, the students are given tasks requiring increasing amounts of responsibility, which culminates in a period of nearly full autonomy (Cameron, 1997). A major drawback of this model, however, is the lack of objectivity in the assessment of the technical skills of a resident. Moreover, it has been difficult to link between the technical skills acquired during residency and surgical outcomes (Darzi et al., 1999). In an in-depth review, Meier et al. (2001) examined the outcomes of current system of training and showed that there is significant variability in educational experience that can be attributed to the random opportunity of patient flow.

Simulation techniques have been used in tandem with the residency model to improve residents knowledge, surgical skills, and confidence; simulators allow for refining their skills without compromising the patient's right of getting the best care. These simulators vary in the realism from silicone blocks, through animal models and anatomically correct mannequins, to virtual and augmented reality sets.

### 2.1. Mechanical Trainers

**Figure 2:**
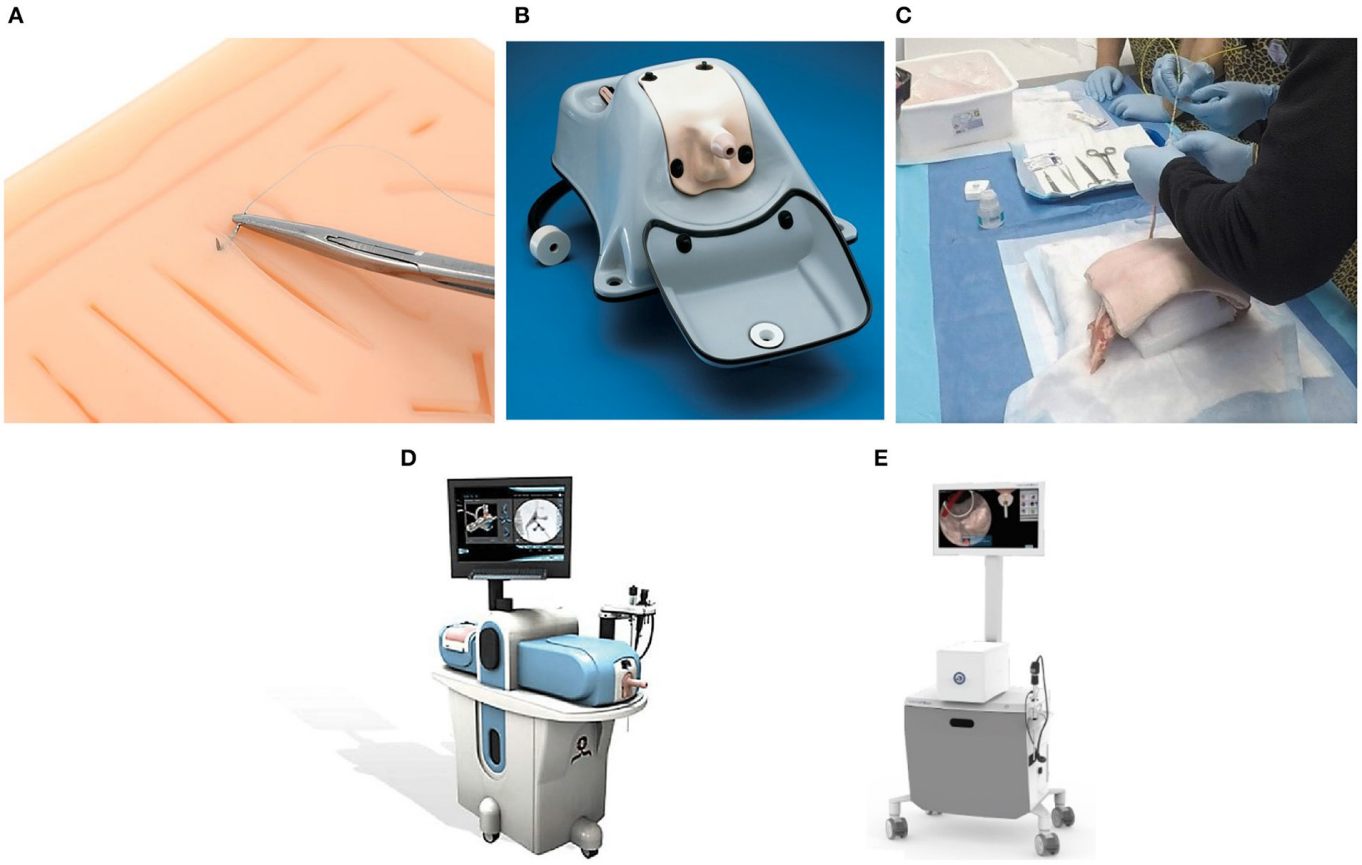
Five types of simulators used to train surgeons: **(A)** A low-fidelity simulator for knot tying by Eisco labs (Source: https://www.eiscolabs.com/products/premium-practice-suture-kit), **(B)** Uro-Scopic Trainer™ which is a high fidelity mechanical simulator for practicing urological procedures (Source: http://assets.limbsandthings.com/documents/Urology_REV_2.pdf), **(C)** an example of PCNL animal lab training, **(D)** Perc Mentor™ (Source: http://simbionix.com/wp-content/pdf/Brochures/healthcare-uro-brochure-2017.8_en-Web.pdf), and **(E)** shows VitraMed UroS™ (Source: https://www.virtamed.com/files/6215/5177/8258/VirtaMed_UroS_Factsheet_EN_V180301.pdf) which is a virtual reality simulator for urological procedures.

Mechanical trainers fall into one of two categories. Low fidelity mechanical trainers help medical students in the development of dexterity. A simple example of a low fidelity simulator is the Eisco Labs module, shown in Figure 2A, which is used to develop skills and dexterity needed for stitching tissue. These trainers give students an opportunity to practice specific manual skills (Hammoud et al., 2008), meaning that they do not need to be anatomically similar. As a result, low fidelity simulators are cost-efficient means of training rudimentary surgical skills; they do not, however, develop student's judgment skills. They also cannot supply objective metrics necessary to judge the skill level of the students.

High fidelity trainers, on the other hand, can be anatomically correct as exemplified by Uro-Scopic™ trainer in Figure 2B. The added anatomic realism and complexity allow students to practice more sophisticated procedures using surgical tools (Hammoud et al., 2008). Uro-Scopic™ trainer, for instance, allows students to remove kidney stones, conduct bladder examinations, and insert stent and guidewire. High fidelity simulators these systems are an effective tool in teaching medical students, but they also have limitations. For instance, the simulator does not afford students the ability to work with all the equipment in the operating room. Since these simulators use physical mannequins, there are only finite unique cases available for student's training. Additionally, the haptic sensations produced by the mannequins may differ significantly from the sensations experienced during surgery. This not only can impact the effectiveness of the training, but it also makes it difficult to determine the skill level of the student.

### 2.2. Animal Labs

Animal labs, an example is shown in Figure 2C, are another type of simulator used to train surgeons. In these labs, animal tissue, typically porcine, is used to build a human analog. Students can interact with the tissue like with a real patient by using surgical tools, and the operating room equipment, if available (Strohmaier and Giese, 2005). Since the tissue originates from an animal it feels realistic to touch. Depending on the construction, however, the analog may not be anatomically accurate. The main concern in maintaining an animal lab is the cost of maintenance. Animal tissues decomposes quickly and a limited number of students can use a single mannequin. As a result, the tissue needs to be replaced frequently increasing the long term costs. Additionally, this training method does not provide an objective and quantifiable insight into the ability level of the students.

### 2.3. PERC Mentor™

The PERC Mentor™ is a hybrid simulator consisting of a physical mannequin model of a human flank and an electronic component simulating the instruments and equipment, as shown in Figure 2D. The users interact with an anatomically correct mannequin which provides the user with the haptic sensations. The user also interacts with real instruments, like nephroscopes and graspers, as well as virtual operating room equipment, like the fluoroscopy imaging which is displayed on the screen. PERC Mentor™ provides objective measures of user performance and it features anatomically correct models. It still relies, however, on physical mannequins which limits the training possibility. This simulator was evaluated by Knudsen et al. (2006) for face, content, and construct validity, and Knudsen et al. (2006), Mishra et al. (2010), and Papatsoris et al. (2012) showed that the simulator is an effective tool in training surgeons.

### 2.4. Virtual Reality Simulators

Virtual reality simulators do not rely on physical objects to provide haptic sensations. Instead, the physical interactions are rendered by a robotic haptic system, while the visuals are shown using either a screen or a head-mounted display. The user interacts with a tool attached to the haptic system which measures the position of the user. Based on the position, a corresponding force output is calculated and then displayed by the haptic system. One example of a VR simulator is the VirtaMed UroS™ shown in Figure 2E. Since the motion of the user is recorded by the haptic system, VR simulators can provide means of objectively measuring the performance of the user. Additionally, because the user does not interact with physical objects, it is possible to include and add multiple anatomical models at a low cost, and with no hardware changes.

Rendering realistic images is simple when provided with adequate computer hardware. It is much more difficult, however, to provide realistic physical sensations. As discussed by Colgate and Schenkel (1997), the quantization of the position data makes haptic devices unstable when rendering high impedance. As a result, there is an intrinsic limit to the impedance generated by a conventional haptic device. Hayward and MacLean (2007) explain the impact of the limited impedance range on the haptic performance, highlighting the inability of haptic devices to generate detailed textures. Therefore, virtual reality simulators can be an effective and robust tool in training surgeons. The primary limitation of a virtual reality simulator is the realism of the haptic feedback. Let us now consider Marion's simulator.

## 3. Marion Surgical K181 PCNL Simulator

**Figure 3:**
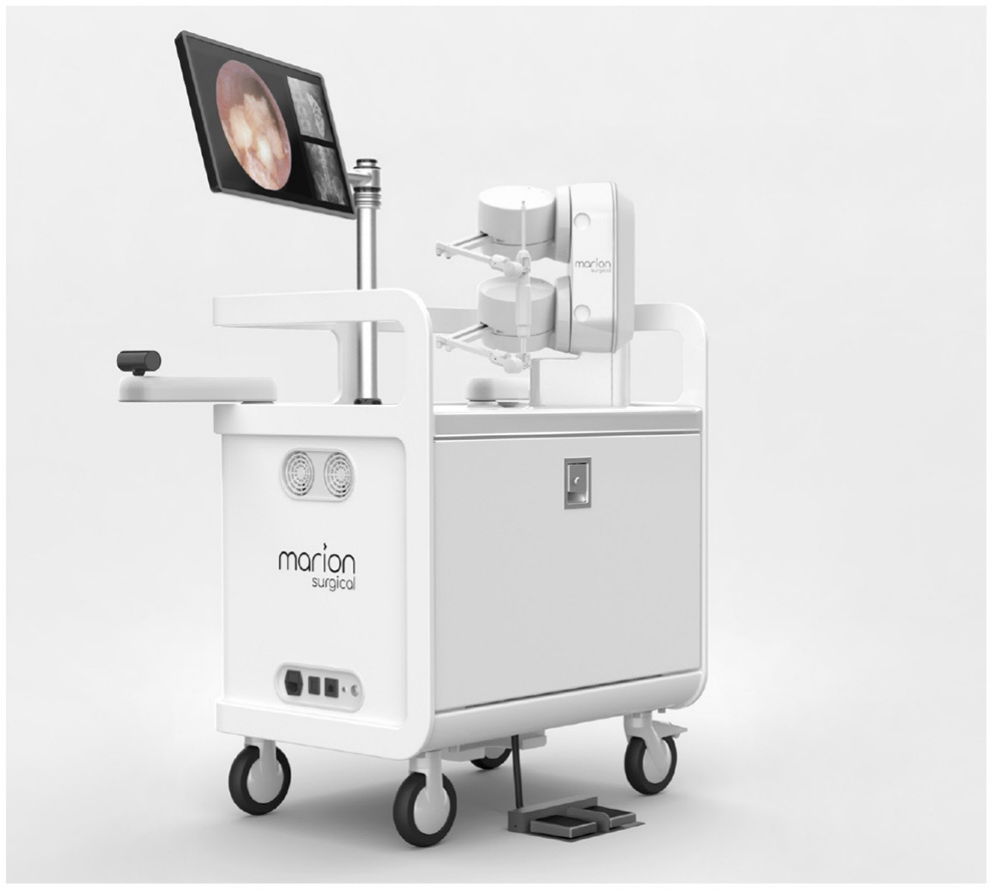
Marion Surgical K181 simulator hardware consists of the enclosure with a screen (for instructor use), a haptic system attached to the case and a VR headset (not shown). The computer, found inside of the enclosure, runs the tissue simulation and renders the virtual operating room.

Marion Surgical K181, shown in Figure 3, is a virtual reality surgical simulator that allows the users to interact with a virtual patient in a virtual operating room. The system has three main components: the virtual reality headset puts the user in a virtual operating room, and the haptic system provides the user with haptic force-feedback calculated by the tissue simulator.

### 3.1. Virtual Reality Operating Room

**Figure 4:**
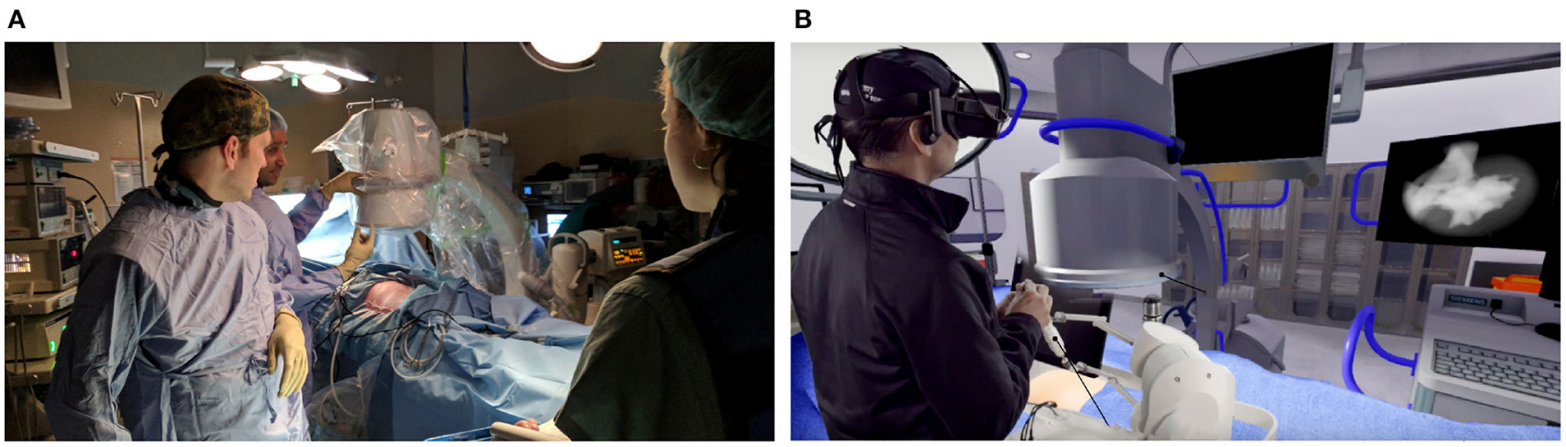
Kidney stone removal operation in a real operating room **(A)** and the simulated surgery in the virtual operating room **(B)**.

The simulator uses the HTC VIVE VR headset to render a virtual operating room, shown in Figure 4B. The headset renders the scene at the resolution of 2,160 × 1,200 and a refresh of 90 Hz providing an immersive and realistic image. The Leap Motion Controller captures the hand location of the user. The position data is then used to show a representation of the user's hands in virtual reality.

The design of the virtual operating room (VROR) was modeled after a real operating theater, like the one shown in Figure 4B, to maximize realism. It includes a C-Arm that is used by surgeons to obtain an image of the kidney which is then displayed on the screen, as shown in Figure 4B. The user can adjust the position and orientation of the C-Arm, using either their hands, via the Leap Motion Controller, or through voice commands. The screen in VROR can also be used to display the video feed from the end of the endoscope.

During the simulation, the user physically interacts with surgical tools like endoscopes, needles, and graspers. Since the VR headset obstructs the user's view, the VROR tools are also represented in the VROR. The motion of these tools is constrained by the range of the haptic devices, which are connected directly to them.

### 3.2. Haptic System

The tools used to physically interact with the patient are connected to 2 Entact W4D force feedback devices which can generate a peak force of 5 N. Each of the devices has 3 active and 3 passive degrees-of-freedom (DOF). The two devices attach to a single tool with 5 active DOF, or to two tools independently with 3 active DOF each.

**Figure 5:**
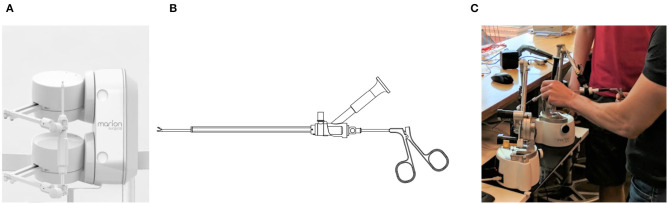
For kidney stone access, the tool connects to the two force-feedback devices, like in **(A)**. The endoscope and grasper tools shown in **(B)** are connected individually to the two haptic robots, as shown in **(C)**, when simulating a kidney stone removal procedure.

To establish kidney access, a surgeon inserts a needle through the skin and into the patient's kidney. To render the force-feedback of the procedure, the needle requires 6 active DOF. The torque due to rotation of the needle, however, is insignificant. By neglecting the rotation of the needle, the required 5 active DOF are provided by two haptic robots attached as shown in Figure 5A.

The kidney stone removal procedure, on the other hand, is completed using two tools shown in Figure 5B, attached to the two robots independently, as shown in Figure 5A. The endoscope provides the surgeon with the view inside of the kidney, while the grasper is used to grab the stones. Each tool connects to one of the robots giving each of them only 3 active DOF without restricting their motion. Note, surgeons insert and remove both tools as a single unit; notice that in Figure 5B the grasper holding the stone does not fit through the opening in the endoscope. Once, the grasper is inserted into the endoscope, the two tools become coupled. As a result, the endoscope is free to move and rotate in any direction while providing 5-DOF force feedback, and the grasper can only move and provide force-feedback in the direction along the channel of the endoscope.

### 3.3. Tissue Simulation

The forces displayed to the user during the simulation are calculated using tissue simulation software, which also renders the fluoroscopic images and the endoscope video feed. The tissue simulation loop runs asynchronously with the haptic and the visual loop, at a refresh rate of 90 Hz. During the procedure, the position and orientation of the tool(s) is recorded in a text file. The positional data is later used to rate the performance of the user.

Since the haptic devices are intrinsically unstable, the simulation uses a virtual coupling to aid in stabilization of the simulated interactions (Colgate et al., 1995; Colgate and Schenkel, 1997). As a result of the coupling, the impedance range of the device is limited and, in extreme cases, there is a noticeable discrepancy between the position of the tools in reality and in the simulation. These are, however, borderline cases involving forces far in excess of what surgeons should use during the procedure.

## 4. Simulator Evaluation

The kidney access and the kidney stone removal modules provided by Marion Surgical K181 simulator were evaluated independently for face and construct validity using 4 experiments summarized in Table 1.

**Table 1:** Summary of the testing including the test type, number of participants and the location of the respective results.

**Module**	**Validation type**	**Number of participants**	**Results**
Kidney stone removal	Face	12	Table 2
Kidney Access	Face	20	Table 3
Kidney stone removal	Construct	14	Table 4
Kidney Access	Construct	20	Table 5

**Table 2:** The results of the questionnaire for the kidney stone removal module showing the mean and the standard deviation (SD) of the scores.

**Metrics**		**Mean (*SD*)**
Realism	Visual realism	3.86 (0.0693)
	Force feedback	3.86 (0.479)
	Instrument movement	3.93 (0.564)
	Anatomic realism	3.86 (0.714)
Usefulness	Compared with traditional	4.76 (0.464)
	Usefulness for teaching	4.41 (0.537)
	Usefulness for assessing	4.05 (0.876)
	Comfort	4.46 (0.621)
	Overall Rating	78.3% (11.9)

### 4.1. Face Validation

Face validation establishes whether a simulator looks and feels realistic to the users, according to Carter et al. (2005). To establish face validity of Marion's simulator the kidney access and kidney stone removal modules were tested independently by two groups comprised of experts and novices.

#### 4.1.1. Methodology

First, a group of 12 subjects including, 1 medical student, 1 resident, 2 clinical fellows, and 8 staff surgeons were recruited to complete a simulation where they had to remove 10 kidney stones. The second group of 20 participants, formed of 14 residents, 2 interventional radiologists, and 4 attending urologists, on the other hand, was tasked with establishing kidney access in the lower pole calyx. After the experiment, each participant completed a questionnaire where they could rate various aspects of the simulator on a 5-point Likert scale. In the questionnaire, the participants were asked to report their opinions about the quality of the simulation. This includes the realism of the target objects from the images fed into the system and instrument handling, the usefulness of the system in practicing the procedure and the overall usability of the system. The questions focused on the accuracy of the simulated procedure and the realism of the virtual experience as compared to the traditional training methods.

Based on the responses of each group, the mean and standard deviation of the participant responses was calculated and shown in Table 3 for the kidney access module, and Table 2 for the kidney stone removal module. The *p*-value was calculated to determine if the difference between the expert and novice users was statistically significant; the null hypothesis states that there is no difference between expert and novice responses.

**Table 3:** The questionnaire results for participant of the PCNL access module evaluation.

**Metric**		**Mean (*****SD*****)**			
		**Total (*n* = 20)**	**Novices (*n* = 17)**	**Experts (*n* = 3)**	***p***
Haptic Realism	Realism of instrument handle	2.7 (1.45)	2.6 (1.50)	3.3 (0.943)	0.57
	Realism of the targets	3.4 (1.49)	3.2 (1.54)	4.3 (0.471)	0.24
	Realism of the haptic feedback	2.4 (1.39)	2.2 (1.38)	3.3 (0.943)	0.20
	Realism (overall)	3.25 (1.51)	3.3 (1.52)	3.0 (1.41)	0.77
Usefulness	Hand-eye coordination training	3.8 (1.38)	3.7 (1.45)	4.3 (0.471)	0.49
	Rehearsal tool	3.8 (1.37)	3.6 (1.45)	4.3 (0.471)	0.44
	Likelihood to recommend	3.6 (1.56)	3.5 (1.65)	4.3 (.0471)	0.40
Image Quality	Quality	4.1 (0.624)	4.1 (0.639)	4.3 (0.471)	0.51
	Brightness	4.3 (0.781)	4.3 (0.823)	4.3 (0.471)	0.94
	Resolution	3.95 (0.920)	3.9 (0.963)	4.3 (0.471)	0.46
	Delay in image display	4.05 (0.920)	4.1 (0.937)	4.0 (0.816)	0.92
	Comfort	3.9 (1.04)	3.9 (1.08)	4.0 (0.816)	0.87
	Realistic (yes)	95%	94.1%	100%	

#### 4.1.2. Results

The kidney access procedure was rated as generally realistic by 95% of the participants, which included all 3 experts. The results showed that the experts found the simulator more realistic than novices. In particular, the experts positively rated the realism of the target and the haptic feedback, compared with the novices, who were more negative in their assessment. The *p*-value was relatively high, 0.24 and 0.20, respectively, for both target and haptic feedback realism, therefore further testing is required to reject the null hypothesis. Experts and novices favorably rated the visual realism. In general, both groups agreed that the image quality, resolution, and brightness of the VR headset were satisfactory. The users also agreed that the VR headset was comfortable, and the delay in image response was minimal. A high *p*-value suggests that there may not be a difference in the assessment of the two groups. Finally, the experts agreed that the simulator is a useful tool for hand-eye coordination training and preparation for surgery.

The kidney stone removal was rated positively, at an average of 78.3%, by the participants of the study. The users found the simulator visuals, haptic feedback, instrument movement, and anatomy to be realistic, rating it around 3.9. The users agreed that the simulator was a beneficial addition for teaching (4.41) and assessing (4.05) the urological skills. In addition, the participants indicated that K181 had benefits over traditional training methods.

### 4.2. Construct Validation

To establish construct validity, according to Carter et al. (2005), the simulator must distinguish between novice and expert users. To this end, the two modules of the simulator were tested by two groups comprised of novices and experts.

#### 4.2.1. Methodology

First, a group of 14 participants, comprised of 1 undergraduate student, 4 medical students, 1 post-doctoral research fellow, 5 residents, 2 clinical fellows, and 1 faculty member, completed a simulation where 10 stones had to be removed from the kidney. On the other hand, a group of 20 participants, including 14 residents, 2 international radiologists, and 4 attending urologists, was tasked with establishing kidney access in the lower pole calyx. Prior to the experiments, each user was introduced to the simulator and given an opportunity to interact with VR and the haptic system. During the experiment the tool path and tool orientation was recorded.

The collected data was normalized by subtracting each feature by mean and dividing by the standard deviation. Descriptive statistics were used to compare the mean value of different kinematic features between expert and novice surgeons. Logistic Regression with forward entry in the training data was applied for the prediction of expertise class. Cohen's d effect size (ES) was also used to estimate the magnitude of the difference between the two groups. According to Cohen, ES of 0.2 to 0.5 is considered small, ES of 0.5 to 1.0 is considered moderate, and ES of greater than 1.0 is considered substantial. The resulting data for the kidney stone removal module test and the kidney stone access module are shown in Tables 4, 5 respectively.

**Table 4:** Analysis of the tool path data collected during the kidney stone removal testing.

**Feature**	**Mean (*****SD*****)**		***p***
	**Novices (*n* = 8)**	**Experts (*n* = 6)**	
Cumulative task time (s)	85.43 (28.97)	59.56 (41.66)	0.20
Path length (m)	3.61 (1.39)	2.06 (1.97)	0.034
Mean Velocity (m/s)	0.05 (0.01)	0.04 (0.03)	0.38
Velocity variance (m^2^/s^2^)	0.04 (0.02)	0.01 (0.05)	0.097
Mean x orientation (deg)	316.63 (27.13)	330.98 (53.89)	0.35
Mean y orientation (deg)	323.55 (23.07)	314.08 (27.1)	0.53
Mean z orientation (deg)	143.64 (80.38)	223.79 (82.03)	0.073
x orientation variance (deg^2^)	10232.09 (5811.34)	7562.37 (7542.65)	0.47
y orientation variance (deg^2^)	7898.49 (6232.55)	10530.08 (5684.53)	0.51
z orientation variance (deg^2^)	11886.43 (10055.88)	6490.5 (6411.89)	0.26

**Table 5:** Analysis of the tool path data gather during the simulation of PCNL kidney access procedure.

**Feature**	**Mean (SD)**		***p***
	**Novices (*n* = 17)**	**Experts (*n* = 3)**	
Cumulative task time (s)	174 (108)	214 (13.9)	0.55
Path length (m)	2.75 (1.35)	2.61 (0.459)	0.87
Mean velocity (mm/s)	0.200 (7.5 × 10^-3^)	0.15 (4.9 × 10^-3^)	0.36

#### 4.2.2. Results

During the simulation, expert surgeons completed the stone extraction with a shorter path length compared to novice surgeons (2.06 m vs. 3.61 m, ES = 0.90, *p* = 0.034). Expert surgeons finished the task in less time compared to novice surgeons (ES = 0.7, *p* = 0.20), despite no difference in instrument motion velocity between the two groups (ES = 0.40, *p* = 0.38).

The same correlation could not be established for the kidney access procedure. The reason for this discrepancy may stem from the different amount of motion required to complete each procedure. Accessing the kidney is an open-ended problem requiring judgment skills; users can use various methods and access points to accomplish it. Each of these methods can be equally valid and lead to similar end results, but they may take different amounts of time and require shorter or longer movements. Additionally, the majority of user motion, in the kidney access procedure, takes place during preparation for the puncture. The amount of time and the travel distance of the needle during the puncture, on the other hand, is relatively short. In kidney stone removal task, on the other hand, the user must repetitively insert the tools into a single opening. A separate set of metrics and a higher number of participants may aid in establishing the construct validity for the kidney access module.

## 5. Discussion

According to Våpenstad et al. (2013a,b, 2017), the transparency of a haptic device plays a key role in VR surgical simulation. The transparency of Marion's simulator might be improved by replacing electric motors with brakes. Magnetorheological brakes can generate higher torque per unit of mass or volume than a conventional electric motor, as shown by Rossa et al. (2013a). A passive haptic device, therefore, will be able to generate a wider range of impedance without risk of instability. However, as shown by Lacki and Rossa (2019), developing a 5-DOF passive haptic system is bound to cause control difficulties. Alternatively, instead of replacing the electric motors from the device, the brakes can be integrated into the existing design. As demonstrated by Rossa et al. (2013b), using an electric motor in tandem with an electric brake results in a stable haptic interaction.

To evaluate construct validity, we attempted to distinguish the experts and novices based only on their tool path. The results showed that during the kidney stone removal procedure, the skill level of a user was correlated to the tool path length; Experienced surgeons completed the procedures with more efficient movements than the novices. For the kidney access procedure, however, there was no correlation between the motion of the user and their experience level.

Note, that the lack of correlation in the kidney stone access procedure may be a limitation of the data analysis, not necessarily the simulator. With more expert users it may be possible to find a correlation between the path statistics and the skill level of the user. Additionally, there are other metrics that can be used in skill evaluation. The location of the kidney puncture may be an effective measure of the user's dexterity and judgment skills. Hannaford and Sinanan (1999), on the other hand, showed that the force data can be useful in determining the skill level of a user. Collecting the force and torque data during the procedure should, therefore, improve the ability of the system to assess the skill level of the users. There are also other methods that can be used to evaluate the tool path data. By collecting more tools paths from experienced surgeons it may be possible to use a trained neural network, like one discussed by Pao (1989), to determine the skill level of the user. This solution may be difficult to implement, however, due to the task difficulty and number of trials required to train the network, similar to the issues highlighted by Mazurowski et al. (2008).

## 6. Conclusions

Marion Surgical K181 is a virtual reality surgical simulator used to train medical residents in Percutaneous Nephrolithotomy procedures, namely kidney stone access, and kidney stone removal. In this paper, we conducted face and construct validity testing of the simulator based on feedback and performance of experienced urologists and medical students. To test the face validity of the simulator participants were asked to rate the realism of the simulator. The participants indicated that Marion Surgical K181 is an effective tool for surgery rehearsal, hand-eye coordination training, and that the simulator provides benefits over traditional training methods. Based on the scores, Marion K181 can benefit from improvements in haptic realism.

Based on the feedback from the users, Marion Surgical K181 simulator is a useful tool in training urologists. Based on the questionnaire results, the simulator can be added as another tool available to residents aiding in their skill development. We plan to continue developing the system, with a focus on the haptic feedback and user skill classification. Concurrently, we are running a larger follow-on study with more participants, more detailed skill level classifications, and a more sophisticated algorithm for analyzing the user performance, which we will use to reinforce the findings of this study.

## Data Availability Statement

All datasets generated for this study are included in the article/supplementary material.

## Author Contributions

BS lead a team constructing the Marion Surgical K181 simulator. Also, BS recruited a team of specialists to conduct the testing of the simulator. The team composed of MS, MGo, AB, LC, JL, and TA developed the testing procedure, tested the simulator, and conducted the analysis of the data independently of the other authors. BS, MŁ, and MS wrote the manuscript based on the findings of the team of specialists. Finally, MŁ, JR, MGr, and CR edited the manuscript.

### Conflict of Interest

BS and MŁ were part of the team that developed the PCNL K181 surgical simulator. The evaluation of the simulator was conducted by surgeons and residents at arms length from the authors. The remaining authors declare that the research was conducted in the absence of any commercial or financial relationships that could be construed as a potential conflict of interest.
